# Deficiency of kin17 Facilitates Apoptosis of Cervical Cancer Cells by Modulating Caspase 3, PARP, and Bcl-2 Family Proteins

**DOI:** 10.1155/2022/3156968

**Published:** 2022-07-20

**Authors:** Bingsen Su, Meifeng Zhong, Yuzhao Zhang, Kunhe Wu, Qiyuan Huang, Chuiyu Zhu, Tao Zeng

**Affiliations:** ^1^Zhongshan Torch Development Zone People's Hospital, Zhongshan, Guangdong 528437, China; ^2^Department of Medical Laboratory, Affiliated Hospital of Guangdong Medical University, Zhanjiang, Guangdong 524000, China; ^3^Department of Laboratory Medicine, The First Affiliated Hospital of Guangzhou Medical University, Guangzhou, Guangdong 510120, China; ^4^Department of Laboratory Medicine, The First Affiliated Hospital, Guangzhou University of Chinese Medicine, Guangzhou, Guangdong 510405, China; ^5^Department of Pathology, Guangdong Women and Children's Hospital, Guangzhou, Guangdong 511400, China; ^6^Department of Clinical Biobank Center, Zhujiang Hospital, Southern Medical University, Guangzhou, Guangdong 510515, China

## Abstract

**Background:**

The treatment of cervical cancer in the late stage is still quite challenging, because of nonspecificity in conventional therapies and the lack of molecular targeted drugs. It is necessary to find novel biomarkers for cervical cancer treatment.

**Methods:**

In the present study, cervical cell lines HeLa and SiHa with kin17 knockdown were constructed by transfection of the recombinant lentiviral vector carrying KIN17 siRNA and screened by puromycin. The established cells with kin17 knockdown were determined by fluorescence observation and western blotting. Cell apoptosis and the mitochondrial membrane potential (MMP) were detected by flow cytometry. The activity of caspase 3 enzyme was tested by spectrophotometry. The expression profile of apoptosis-associated proteins was analyzed by western blotting. Finally, we used bioinformatics and proteomic data to analyze KIN-related genes in cervical cancer.

**Results:**

The results showed high fluorescent positive rates (>90%) and high gene silencing efficiency (>65%) in HeLa and SiHa cells transfected with gene silencing vectors. Moreover, kin17 deficiency decreased the MMP and increased the apoptosis rates in HeLa and SiHa cells, respectively. Furthermore, knockdown of kin17 enhanced the activity of caspase 3 enzyme, increased the expression of cleaved PARP and Bim, while decreasing the expression of Bcl-xL and phosphorylated BAD in HeLa and SiHa cells. Identification of KIN-related prognostic genes in cervical cancer revealed that a total of 5 genes (FZR1, IMPDH1, GPKOW, XPA, and DDX39A) were constructed for this risk score, and the results showed that CTLA4 expressions were negatively correlated with the risk score.

**Conclusion:**

Our findings demonstrated that kin17 knockdown facilitates apoptosis of cervical cancer cells by targeting caspase 3, PARP, and Bcl-2 family proteins. Besides, kin17 could regulate cancer cell apoptosis through the mitochondrial pathway and could be used as a novel therapeutic target for the regulation of cell apoptosis in cervical cancer.

## 1. Introduction

Cervical cancer is the third most common malignancy and the fourth leading cause of cancer-related death in women worldwide [1, 2]. Cancer has been linked with the persistent occurrence of high-risk human papillomavirus (HPV) infection, an extremely common virus transmitted through sexual contact. Although the burden of cervical cancer has declined in different regions due to effectively implemented screening, it remains a major concern in low- and middle-income countries [3]. Cervical cancer in the early stage is always asymptomatic, while conventional therapies fail to control cervical cancer in the late stage. Besides, molecular targeted therapies have been developed in the treatment of cervical cancer, while the unsatisfactory performance of molecular targeted drugs for the cancer was displayed in clinical trials [4]. The induction of cancer cell apoptosis is recognized as one of the key strategies for cancer therapy. Recent studies have shown that inhibited cell apoptosis is related to the development of cervical cancer [5]. However, the mechanisms involved in cervical cancer cell apoptosis remain unclear and should be elucidated to develop new therapeutic targets.

In 1989, the KIN17 gene was first identified by Angulo et al. Kin17 is an extremely conserved protein in the evolutionary process of the species, which has been identified with antibodies directed against the bacterial RecA protein. Also, it is generally expressed in the heart, testis, and skeletal muscle cells, with lower expression in other organs [6]; it has been found to be involved in a variety of important physiological and pathological processes, including DNA replication and transcription, DNA damage response to ionizing radiation, immunoglobulin isotype switching, and tumor cell proliferation in different cancer types [7]. Recent studies have suggested that kin17 is highly expressed in a variety of malignant tumors, such as breast cancer, lung cancer, liver cancer, and melanoma, and it is closely related to tumorigenesis and progression of these tumors [8–11]. Hence, kin17 has been regarded as a novel biomarker and target for diagnosis and treatment of some cancers.

Interestingly, we associated kin17 with the apoptosis of breast cancer cells in our previously published works [12]. Previous studies by our group also showed that kin17 was obviously upregulated in tissue samples from patients with cervical cancer [13]. Yet, the association between kin17 and cervical cancer cell apoptosis remains unknown. In the present study, we examined the roles and mechanisms of kin17 in the apoptosis of cervical cancer cells to assess the application potential of kin17 in the treatment.

## 2. Materials and Methods

### 2.1. Cell Culture

Human cervical cancer cell lines HeLa (adenocarcinoma cells; cat. No. TCHu187; GeneChem, Shanghai, China) and SiHa (squamous carcinoma cells; cat. No. TCHu113; GeneChem, Shanghai, China) were cultured in Dulbecco's modified eagle medium (DMEM; Gibco, Carlsbad, CA, USA) supplemented with 10% fetal bovine serum (FBS; Tianjin Kangyuan Biotechnology, Tianjin, China) and 1% antibiotics (Hyclone, USA) consisting of 100 *μ*g/mL penicillin and streptomycin. The cells were incubated in 5% CO_2_ and 95% humidity at 37°C.

### 2.2. Cell Transfection and Cell Line Screening

The lentiviral vector GV248 (GeneChem, Shanghai, China) containing a reporter gene enhanced green fluorescent protein (EGFP) gene was used for gene modification. The gene-silencing lentiviral vector GV248-KD with a specific siRNA-targeting KIN17 gene sequence and the normal controlled lentiviral vector GV248-NC were successfully constructed, as previously described [8]. HeLa and SiHa cells were seeded into 6-well plates at a density of 1.3 × 10^5^ cells/well and transfected with the lentiviral vector GV248-KD (HeLa^KD^, SiHa^KD^ cells) or the vector GV248-NC (HeLa^NC^, SiHa^NC^ cells) using Opti-MEM (Gibco, Thermo Fisher Scientific, Inc.) and polybrene (Shanghai GeneChem, Shanghai, China) following the manufacturer's protocol. The volumes of the lentiviral vectors were calculated according to the formula provided and the final concentration of polybrene (5 *μ*g/mL) recommended by the manufacturer. Then, the transfected cells were cultured in a medium containing 1.5 *μ*g/mL puromycin for screening until the cells reached ∼90% confluence with positive EGFP expression. The effect of kin17 knockdown was assessed by western Blot assay. HeLa cells or SiHa cells without transfection with a vector (HeLa^Mock^ and SiHa^Mock^ cells) were used as blank control.

### 2.3. Mitochondrial Membrane Potential (MMP) Measurement

Cells were digested with 0.25% trypsin and incubated with JC-1 probes (Beyotime, Shanghai, China) at 37°C for 20 min. After being washed with JC-1 1 × buffer twice, the cells were suspended in 500 *μ*L buffer. The green fluorescence of JC-1 monomers (an indicator of a low MMP) and red fluorescence of aggregated JC-1 (an indicator of a high MMP) were captured by using a flow cytometer (BD LSRFortessa™; BD Biosciences, San Jose, CA, USA). In addition, the relative ratios of green fluorescence/red fluorescence were calculated to evaluate the mitochondrial membrane potential in cells.

### 2.4. Cell Apoptosis Measurement

Cell apoptosis was detected by flow cytometry. Briefly, after being harvested and washed with PBS (Gibco; Thermo Fisher Scientific, Inc.), 1 × 10^5^ cells were resuspended with a binding buffer in a tube and then stained with annexin V-APC (BD Biosciences, San Jose, CA, USA) for 15 min at room temperature, according to the manufacturer's protocol. The rates of the cell apoptotic phases were determined by using a flow cytometer (BD LSRFortessa™; BD Biosciences, San Jose, CA, USA).

### 2.5. Caspase 3 Activity Assay

Caspase 3 activity was tested according to the instructions of the detection kit (Beyotime, Shanghai, China). Activated caspase 3 could transform its specific subtract Ac-DEVD-pNA (Acetyl-Asp-Glu-Val-Asp p-nitroanilide) into p-nitroanilide, which has a maximum absorption peak at 405 nm. Hence, 2 × 10^6^ cells were collected in a tube, and then, 100 *μ*L lysis buffer was added into the tube and incubated for 15 min in an ice environment. The supernatant was obtained after centrifugation at a speed of 20000 × *g* at 4°C for 15 min. Finally, the supernatant was incubated with Ac-DEVD-pNA at 37°C for 2 h, and the absorbance of 405 nm was measured at once.

### 2.6. Western Blot Assay

To assess the content of the proteins in the cells, RIPA lysis buffer (Beyotime Institute of Biotechnology, Haimen, China) containing a complete protease inhibitor cocktail tablet (Roche Applied Science, Penzberg, Germany) was used to extract total proteins from the cells as previously described [9]. Then, the total 100 *μ*g of proteins were separated by 12% SDS-PAGE and transferred onto Immobilon®-P PVDF transfer membranes (EMD Millipore, Billerica, MA, USA). Following blocking with nonfat milk at room temperature for 1 h, the membranes were probed with anti-Kin17 (dilution, 1 : 500; cat. No. sc-32769; Santa Cruz Biotechnology, Inc.), anti-Bcl-xL (dilution, 1 : 1000; ^#^2764; Cell Signaling Technology, Inc.), anti-Bim (dilution, 1 : 1000; ^#^2933; Cell Signaling Technology, Inc.), anti-PARP (dilution, 1 : 1000; ^#^9542; Cell Signaling Technology, Inc.), anticleaved PARP (dilution, 1 : 1000; ^#^5625; Cell Signaling Technology, Inc.), anti-BAD (dilution, 1 : 1000; ^#^9239; Cell Signaling Technology, Inc.), anti-p-BAD (dilution, 1 : 1000; ^#^5284; Cell Signaling Technology, Inc.), and anti-GAPDH (dilution, 1 : 500; sc-47778; Santa Cruz Biotechnology, Inc.) monoclonal primary antibodies overnight at 4°C. After washing three times with TBST, HRP-conjugated secondary antibodies (dilution, 1 : 500; cat. No. sc-2318; Santa Cruz Biotechnology, Inc.) were used to develop immunoblots at room temperature for 1 h, which were processed using ab ECL-enhanced chemiluminescence substrate (Thermo Fisher Scientific, Inc.). Images were captured using the ImageQuant RT ECL™ imager (GE Healthcare Life Sciences, Shanghai, China).

### 2.7. Bioinformatics and Proteomics Data Processing

In addition, to analyze significantly expressed differential proteins, 306 cervical cancer tumor samples were collected from TCGA database, 70% of which were training and 30% validation, and the comparison of each distinct difference was analyzed using the ingenuity pathway analysis tool (IPA, Qiagen, Hilden, Germany) for gene ontology and functional enrichment analysis. Then, the expression data were used to estimate stromal and immune cells in malignant tumor tissue. The stromal and immune scores generated by the ESTIMATE algorithm were used to estimate the levels and tumor purity of infiltrating stromal and immune cells in cervical cancer tissue by expression profiling. Deconvolution results of tumor-infiltrating immune components were generated from the data collected from TCGA database, which was analyzed by the MCP-counter algorithm. To predict SKCM response to cancer therapy by immune checkpoint blockade, we used the TIDE database (https://tide.dfci.harvard.edu) to test how gene expression in cervical cancer correlates with high levels of cytotoxic T lymphocytes horizontal infiltration interactions affect patient survival.

### 2.8. Statistical Analysis

All experiments were repeated in three independent experiments, and the data were presented as mean ± standard deviation (SD). Statistical product and service solutions (SPSS) software (version 16.0; SPSS, Inc., Chicago, IL, USA) was used for all statistical analyses. The normality test and homogeneity test of variance were performed to clarify the type of data. The independent Student's *t*-test was used to analyze the difference among the two groups and one-way ANOVA (LSD) was performed to analyze the difference among the three groups when parametric data met normal distribution and homogeneity of variance. All statistical results and corresponding *P* values reported were two-tailed. *P*-value of <0.05 was considered a statistically significant difference.

## 3. Results

### 3.1. Cervical Cell Lines HeLa and SiHa with kin17 Knockdown were Established

**Figure 1:**
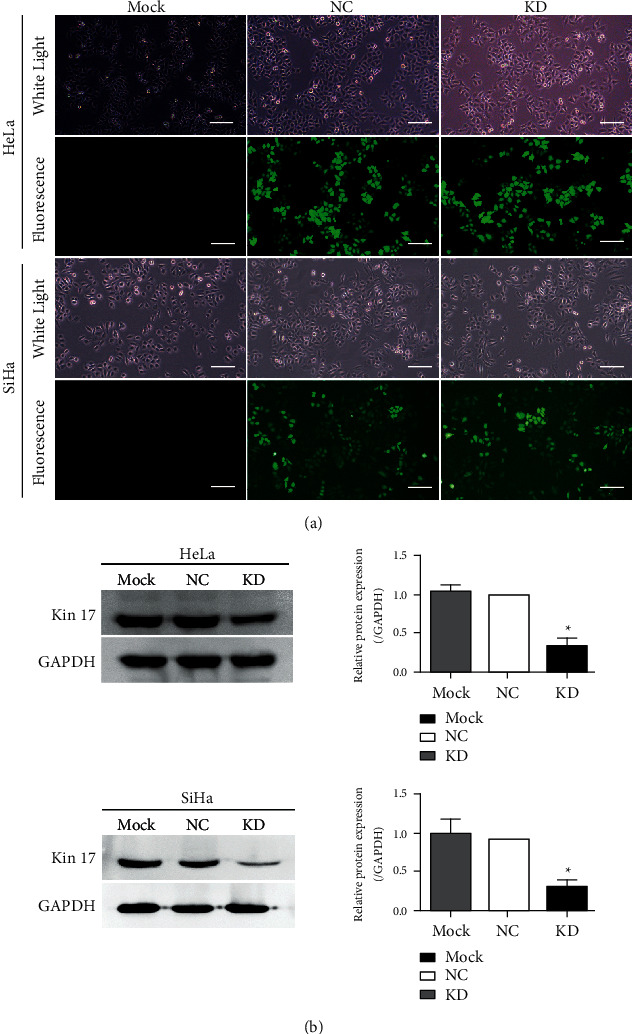
Establishment and determination of cervical cells line HeLa and SiHa with kin17 knockdown. (a) morphological features and fluorescence-indicated infection of HeLa^Mock^, HeLa^NC^, HeLa^KD^ cells, SiHa^Mock^, SiHa^NC,^ and SiHa^KD^ cells, ×100. Scale bars = 200 *μ*m. (b) protein levels of kin17 in HeLa cells and SiHa cells transfected with lentivirus were identified by western blotting, and the densitometric quantification was showed. HeLa^KD^, HeLa cells transfected with recombinant lentiviral vectors carrying the siRNA-targeting KIN17 gene; HeLa^NC^, HeLa cells were transfected with the control vector; HeLa^Mock^, HeLa cells without transfection of the vector. SiHa^KD^, SiHa cells infected with recombinant lentiviral vectors carrying the siRNA-targeting KIN17 gene; SiHa^NC^, SiHa cells transfected with the controlled vector; SiHa^Mock^, SiHa cells without transfection of the vector. NC, negative control; KD, knockdown; ^*∗*^*P* < 0.05, *n* = 3.

To investigate the influence of kin17 in cell apoptosis, the recombinant lentiviral vector with or without KIN17 siRNA was used to transfect HeLa and SiHa cells, after which the stable expression cell colonies were screened by puromycin for nearly a month. The positive fluorescent rates of HeLa^NC^, HeLa^KD^, SiHa^NC,^ and SiHa^KD^ cells were more than 90%, while almost no fluorescence was observed in both HeLa ^Mock^ and SiHa^Mock^ cells (Figure 1(a)). Thus, the high transfection efficiency of the lentiviral vectors was obtained in the two cervical cancer cell lines.

Compared with the HeLa^NC^ cells and SiHa^NC^ cells, the levels of kin17 protein in HeLa^KD^ cells and SiHa^KD^ cells (Figure 1(b)) were dramatically reduced by more than 65% in western blot analysis, thus suggesting the availability of the cervical cell lines HeLa and SiHa established in loss-of-function experiments of follow-up researches.

### 3.2. Knockdown of kin17 Promoted Apoptosis of HeLa and SiHa Cells

**Figure 2:**
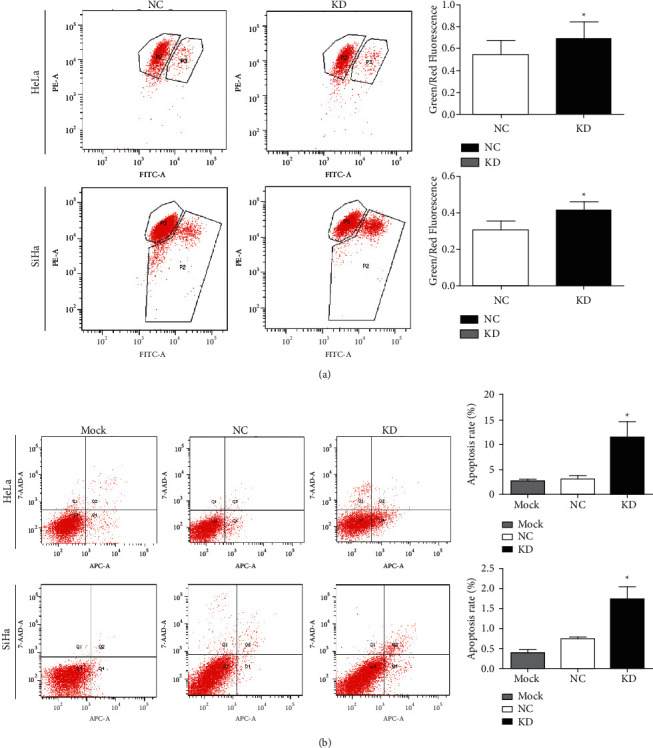
Effect of kin17 knockdown on apoptosis of HeLa and SiHa cells. apoptosis rates of HeLa and SiHa cells with kin17 knockdown and their normal controlled cells were analyzed by flow cytometry (a) representative dot plots with APC fluorescence area (APC-A) as the horizontal ordinate and cumulative data shown, ^*∗*^*P* < 0.05, *n* = 3. relative ratios of green/red fluorescence indicating mitochondrial membrane potential of HeLa and SiHa cells were analyzed by flow cytometry (b) representative dot plots with fluorescence and quantification. NC, negative control; KD, knockdown; ^*∗*^*P* < 0.05, *n* = 3.

Flow cytometry was performed to measure the apoptosis rates and mitochondrial membrane potential of HeLa and SiHa cells after kin17 knockdown. In addition, the relative ratios of green fluorescence/red fluorescence in HeLa^KD^ cells and SiHa^KD^ cells were elevated compared with those in the controlled cells (*P* < 0.05, Figure 2(a)), thus suggesting the decrease of the mitochondrial membrane potential by kin17 deficiency. Compared with the normal controlled cells, higher rates of cell apoptosis were observed in HeLa^KD^ cells and SiHa^KD^ cells (*P* < 0.05, Figure 2(b)), respectively.

### 3.3. Knockdown of kin17 Changed the Expression Profile of Apoptosis-Associated Proteins in HeLa and SiHa Cells

**Figure 3:**
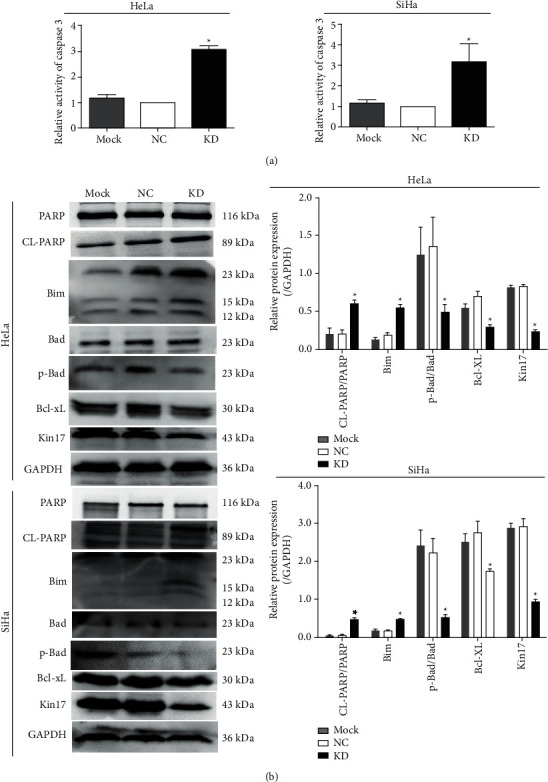
Effect of kin17 knockdown on the expression profile of apoptosis-associated proteins in cervical cancer cells. activities of caspase 3 in kin17^KD^ and kin17^Mock^ cells was normalized to the kin17^NC^ cells (a), the average value of which was set as 100%, ^*∗*^*P* < 0.05, *n* = 3. total-length PARP, cleaved PARP, Bim, Bcl-xL, bad, phosphorylated bad in HeLa and SiHa cells were detected via western blotting (b). NC, negative control; KD, knockdown; PARP, poly ADP-ribose polymerase; Bim, Bcl-2 interacting mediator of cell death; Bcl-xL, B-cell lymphoma-xL; Bad, Bcl2 associated death promoter.

**Figure 4:**
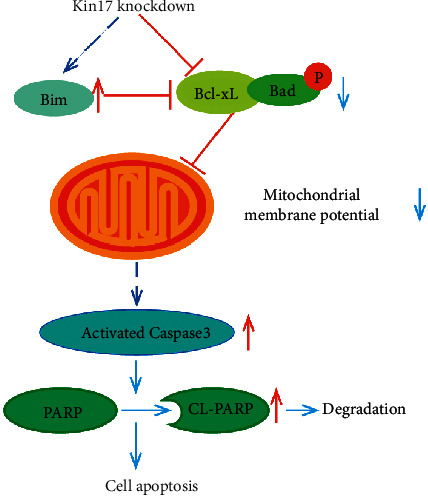
The potential mechanism diagram of kin17 knockdown involved in cervical cancer cell apoptosis. PARP, poly ADP-ribose polymerase; CL-PARP, cleaved poly ADP-ribose polymerase; Bim, Bcl-2 interacting mediator of cell death; Bcl-xL, B-cell lymphoma-xL; Bad, Bcl2 associated death promoter.

HeLa^KD^ and SiHa^KD^ cells showed increased activity of caspase 3 enzyme compared to the normal controlled cells and mock cells (Figure 3(a); *P* < 0.05). The ratio of cleaved PARP/total-length PARP (poly ADP-ribose polymerase) and Bim (Bcl-2 interacting mediator of cell death) were upregulated in HeLa^KD^ and SiHa^KD^ when compared with their corresponding control group in western blot assay (Figure 3(b)). While the ratio of phosphorylated BAD/BAD (poly ADP-ribose polymerase) and Bcl-XL (B-cell lymphoma-xL) was downregulated in HeLa^KD^ and SiHa^KD^ cells (Figure 3(b)). Thus, the mechanism diagram of kin17 knockdown underlying cervical cancer cell apoptosis is shown in Figure 4. Our findings demonstrated that THE knockdown of kin17 promoted apoptosis of cervical cancer cells by targeting caspase 3, PARP, and Bcl-2 family proteins.

### 3.4. Identification of KIN-Related Prognostic Genes in Cervical Cancer

**Figure 5:**
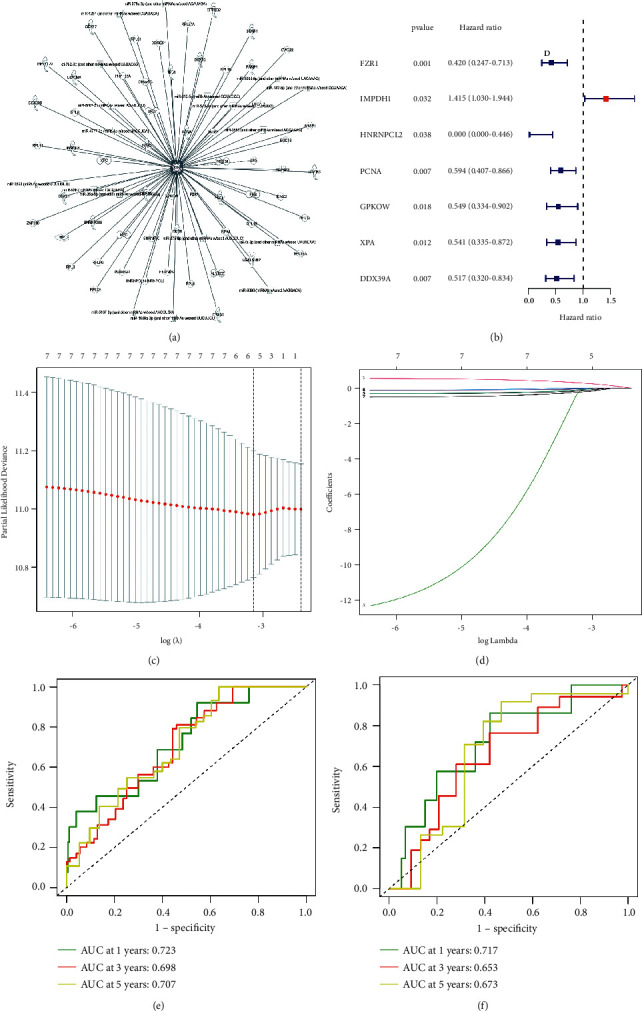
Identification of KIN-related prognostic genes in cervical cancer. (a) graphical summaries of IPA pathway analysis. (b) univariate Cox regression analysis of intersecting genes. ((c)-(d)) LASSO analysis revealing the minimal lambda. ((e)-(f)) time-dependent ROC curve.

We downloaded 306 cervical cancer tumor samples from TCGA, 70% for the training set and 30% for the validation set, and used the IPA database to find KIN-related genes (Figure 5(a)). Univariate Cox regression analysis screened 7 genes (FZR1, IMPDH1, HNRNPCL2, PCNA, GPKOW, XPA, and DDX39A) that were associated with the cervical cancer prognosis (Figure 5(b)). Next, we constructed a risk score of KIN-related genes in cervical cancer. The LASSO regression prognostic model was constructed from 7 genes screened by univariate Cox regression, and finally, a total of 5 genes (FZR1, IMPDH1, GPKOW, XPA, and DDX39A) were constructed in this risk score (Figures 5(c) and 5(d)). Receiver operating characteristic (ROC) curves verified the AUC at 1, 3, and 5 years, respectively (Figures 5(e) and 5(f)).

### 3.5. Association between KIN-Related Genes and Prognosis of Immune Checkpoint Therapy in Cervical Cancer

**Figure 6:**
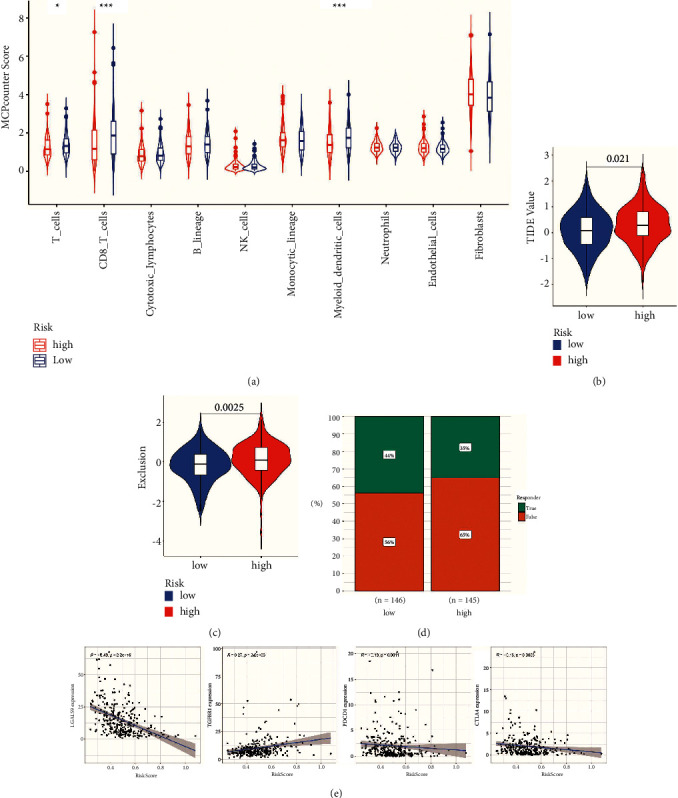
Correlation between prognostic model and immune microenvironment. (a) the infiltrating levels of 10 tumor microenvironments cell types in high/low subtypes in the cervical cancer. ((b)-(c)) violin diagram showing the differential TIDE and TCIA between the high/low-risk groups. (d) chi-squared test plot for immunotherapy in the responder. (e) the expression of four immune checkpoint molecules in two prognostic subtypes.

Furthermore, to determine the relative abundance of tumor-infiltrating immune cells (TIICs) in cervical cancer samples, the degree of infiltration of TIICs was estimated using the MCP-counter algorithm. CD8+ T cells in high-risk patients and high T cells in lower-risk patients (Figure 6(a)). Ultimately, we evaluated the relationship between the risk score and immunotherapy in GC at the TIDE and TCGA (The Cancer Immunome Atlas) (*P* < 0.05, Figures 6(b) and 6(c)). Subsequently, the chi-square plot showed that 44% of the responders in the low-risk group were effective, and 56% were ineffective in the TIDE (tumor immune dysfunction and exclusion); 65% of the responders in the high-risk group were effective, and 35% were ineffective (Figure 6(d)). Intending to ascertain the efficacy of the risk group for immunotherapy, we initially correlated four common immune checkpoints with the risk score. The results showed that CTLA4 expressions were negatively correlated with the risk score (Figure 6(e)).

## 4. Discussion

Apoptosis is a highly regulated form of programmed cell death. In general, there are three major recognized pathways for cell apoptosis (mitochondrial signaling pathway, endoplasmic reticulum pathway, and death receptor pathway) [14]. In addition, the mitochondrial signaling pathway is the most typical pathway for apoptosis. It has been suggested that loss of the mitochondrial membrane potential by apoptotic stimuli in the initialization phase would trigger mitochondria permeability transition, release of caspase-activating proteins, and generation of reactive oxygen species, thus resulting in irreversible cell apoptosis [15, 16]. The present study showed elevated cellular apoptosis rates and a decreased mitochondrial membrane potential after manipulation of kin17, thus indicating the relationship of kin17 with cervical cancer cell apoptosis.

Under normal conditions, caspase enzymes are present in the form of an inactive zymogen, while the activation of caspases enzymes followed by cascade reactions is a vital process of cell apoptosis. Also, caspase 3, known as a “deathase,” is regarded as a key protease in apoptosis [17]. After the activation of caspase-3 by the apoptotic signal, the cell is mainly disintegrated through three mechanisms: enzymatic inactivation of apoptosis inhibitors (I-CAD/DFF45, Bcl-2, and MDM2), enzymolysis of the extracellular matrix and cytoskeletal proteins (actin and laminin), and lysis of DNA repair-associated proteins (PARP) [18–20]. Moreover, PARP is one of the most important substrates for cleavage through the activated caspase 3. Degradation of PARP through cleavage is the key symbol for cell apoptosis by activating endonucleases and enhancing genome instability [21]. It seems that the decreased mitochondrial membrane potential, elevated caspase 3 activity, and increased cleaved PARP by kin17 silencing in cervical cancer cells emphasize the roles of kin17 in the mitochondrial signaling pathway.

Apoptosis acts as a protection mechanism after DNA damage or mutation in an emergency, avoiding tumorigenesis in normal cells. Cervical cancer cells are characterized by sustained DNA replication, accumulated DNA damage, and uncontrolled cell proliferation [22–24]. However, the mechanisms involved in the inhibition of apoptosis in cancer cells remain unclear. In particular, ultraviolet or ionizing radiation triggered upregulation and redistribution of kin17 in cells, thus suggesting the participation of kin17 in DNA damage responses [25, 26]. Evidence also suggests that kin17 is a DNA maintenance protein involved in the cellular response to DNA damage. It also helps to overcome the perturbation of DNA replication produced by unrepaired lesions [27]. Our data demonstrated that knockdown of kin17 led to the degradation of PARP, another DNA repairing enzyme related to DNA damage response and cell survival. It is possible that kin17 plays a crucial role in overcoming the DNA damage checkpoint control and suppressing cell apoptosis through the modulation of PAPR and other DNA repair proteins during tumorigenesis in cervical cancer cells.

The Bcl-2 family of proteins is essential regulators for apoptosis that act by targeting the mitochondrial membrane and stabilizing its potential [28]. In normal cells, Bim acts as a proapoptotic protein by binding to Bcl-2 and Bcl-xL when stimulated by apoptotic signals [29]. Also, BAD with a dephosphorylation form shows proapoptosis activity, whereas phosphorylation could inhibit the proapoptotic activity of BAD by blocking the interaction of BAD with Bcl-2 and Bcl-xL [30]. Our results showed that kin17 knockdown increased the expression of proapoptotic Bim and decreased the expression of antiapoptotic Bcl-xL and phosphorylated BAD in cervical cancer. This suggested that kin17 can regulate cell apoptosis by moderating Bcl-2 family proteins and the mitochondrial membrane potential.

Finally, we used bioinformatics and proteomic data to analyze KIN-related genes in cervical cancer. Identification of KIN-related prognostic genes in cervical cancer revealed that a total of 5 genes (FZR1, IMPDH1, GPKOW, XPA, and DDX39A) were constructed for this risk score, and the results showed that CTLA4 expressions were negatively correlated with the risk score. The high-risk group will have more Treg cell infiltration. Treg cells induce tumors to express more immunosuppressive molecules by inhibiting CD8+ T cells and enabling tumors to produce immune escape. This is consistent with our analysis of immunotherapy. The low-risk group had lower TIDE and higher TCIA scores due to a greater propensity to express the CTLA4 immunosuppressive molecule, suggesting a higher efficacy of immunotherapy in the low-risk group. These findings further elucidate the function of KIN-related genes in cervical cancer and may help us understand the biology of cervical cancer and provide new therapeutic targets. The poor prognosis of cervical cancer appears to depend on a multi-layered relationship between DNA repair gene mutations, cell proliferation, and the interplay of immune responses.

Due to the nonspecificity and side effects related to conventional therapies, molecular targeted drugs have been developed for the treatment of cervical cancer in the late stage. By now, few molecular drugs for cervical cancer showed positive results in clinical trials [31]. Evidence showed that the up-regulation of kin17 was related to several common cancers. Besides, kin17 has been regarded as a promising molecular target for these cancers [8–11, 26]. Besides, it was confirmed that suppressed cell apoptosis was linked to the occurrence and development of cervical cancer [32]. Our previous studies have identified the regulation of kin17 in cell apoptosis of breast cancer and the elevated expression of kin17 in cervical cancer [12, 13]. The present study showed that knockdown of kin17 aggravated the apoptosis degree of cervical adenocarcinoma HeLa cells and squamous carcinoma SiHa cells, thus indicating that kin17 might be a potential target for cancer therapy by inducing cell apoptosis.

In conclusion, our findings demonstrated that knockdown of kin17 promoted the apoptosis of cervical cancer cells by targeting caspase 3, PARP, and Bcl-2 family proteins. Therefore, kin17 might act as a regulator of the cancer cell apoptosis through the mitochondrial pathway and could be used as a novel therapeutic target for regulation of cell apoptosis in cervical cancer. Furthermore, *in vivo* studies and in-depth studies of underlying molecular mechanisms should be performed to elucidate the roles and application prospects of kin17 in cervical cancer.

## Data Availability

The data and materials used in this study are available upon request.
